# Cross‐Sectional Assessment of Medication Adherence and Its Association With Community Pharmacy Experience and Patients' Satisfaction and Characteristics: Findings From the IMPHACT‐LB Project

**DOI:** 10.1002/hsr2.71821

**Published:** 2026-02-22

**Authors:** Fouad Sakr, Chadia Haddad, Jihan Safwan, Aline Hajj, Hala Sacre, Rony M. Zeenny, Marwan Akel, Pascale Salameh

**Affiliations:** ^1^ School of Pharmacy Lebanese International University Beirut Lebanon; ^2^ INSPECT‐LB (Institut National de Santé Publique, d'Épidémiologie Clinique et de Toxicologie‐Liban) Beirut Lebanon; ^3^ Research Department Psychiatric Hospital of the Cross Jal Eddib Lebanon; ^4^ Aging Research Team, Center for Epidemiology and Research in Population Health (CERPOP) Université de Toulouse, Université Paul Sabatier Inserm Toulouse France; ^5^ University Paul Sabatier Toulouse III Toulouse France; ^6^ Faculté de Pharmacie Université Laval Québec Canada; ^7^ Oncology Division CHU de Québec Université Laval Research Center Québec Canada; ^8^ Drug Information Center, Order of Pharmacists of Lebanon Beirut Lebanon; ^9^ Department of Pharmacy American University of Beirut Medical Center Beirut Lebanon; ^10^ School of Medicine Lebanese American University Byblos Lebanon; ^11^ Faculty of Pharmacy Lebanese University Hadath Lebanon; ^12^ Department of Primary Care and Population Health University of Nicosia Medical School Nicosia Cyprus

**Keywords:** community pharmacist, community pharmacy, LMAS‐14, medication adherence, patient satisfaction

## Abstract

**Background and Aims:**

While pharmacists undoubtedly exert considerable influence over medication adherence and the patient experience with healthcare, a complex interplay of factors should also be considered, encompassing the sociodemographic characteristics of patients, the pharmacist‐related factors, patient satisfaction, and the nature of the community pharmacy experience. This study aimed to assess the dynamics of these factors and examine their association with the adherence of patients to medications.

**Methods:**

An online cross‐sectional study was undertaken from April 11 to April 27, 2023, to assess the association between community pharmacy‐related experiences and patient medication adherence in a sample of 865 Lebanese adults using the Lebanese Medication Adherence Scale (LMAS‐14).

**Results:**

Of all participants, 38.5% were regular visitors to the community pharmacy, and 57.7% visited the community pharmacy to obtain nonprescription medications. Almost half of the participants stated that the counseling time by the pharmacist is between 5 and 10 min (47.6%). The majority of the participants perceive the pharmacist as a medication expert (94.2%), a health counselor (81.0%), a health promoter (66.2%), and a practitioner who is patient‐centered (73.3%). Older patient age (Beta = 0.08), always visiting the community pharmacy to obtain nonprescription medications (Beta = 3.63), having public health coverage (Beta = 2.37), and spending more than 10 min on counseling about a medication and/or medical condition (Beta = 3.10) were significantly associated with better medication adherence. Visiting a pharmacy to obtain chronic and nonprescription medications (Beta = −2.98) and having irregular health coverage (Beta = −2.60) were significantly associated with lower medication adherence. However, overall satisfaction with pharmacy experience was not associated with medication adherence (*p* > 0.05).

**Conclusion:**

Pharmacists are seen as experts in medication and holistic healthcare providers. Sociodemographic attributes and specific community pharmacy experience items influence medication adherence. While recognizing that the effects of age and health coverage can guide interventions, emphasizing the role of pharmacists, including extended counseling, could enhance medication adherence and, thus, health outcomes.

## Summary

- What's Known: Pharmacists play an important role in improving medication adherence and patient care, especially in community pharmacy settings.
- What's New: Pharmacists may play a vital role in identifying affordable alternatives and interventions to enhance medication adherence for patients with certain types of insurance that do not cover medications.
- Clinical Implications: Providing patient‐centered care in community pharmacies, such as extended counseling and tailored services, can significantly improve medication adherence, especially in resource‐limited settings. Policymakers and healthcare providers should use these findings to optimize pharmacy services and improve health outcomes in crisis‐affected populations.

## 1 Introduction

Pharmacists have long been recognized as integral healthcare team members, pivotal in ensuring medication safe and effective utilization [1]. With their expertise as medication specialists, pharmacists occupy a unique position, serving as the cornerstone between patients and their prescribed treatments [2]. The specialized training of pharmacists equips them with the knowledge and competencies necessary to optimize medication therapy, diligently monitor for adverse effects, and collaborate seamlessly with other healthcare providers to elevate the quality of patient care [3, 4].

Worldwide, community pharmacies serve as accessible healthcare hubs for patients of all backgrounds. Patients frequently visit these pharmacies for various reasons, ranging from prescription refills to seeking over‐the‐counter medications and health advice [5]. The frequency of patient visits to community pharmacies underscores the opportunity for pharmacists to establish meaningful relationships with their patients. These relations often go beyond dispensing medications, evolving into trusting partnerships where patients feel comfortable seeking guidance on their health concerns [6]. Pharmacists offer a wide array of health services that extend beyond medication dispensing. These services include assisting in self‐care, drug counseling, and health promotion, among other health services [7].

In Lebanon, the role of the community pharmacist has been well described in previous years; this role was particularly highlighted in the current socioeconomic crisis affecting Lebanon, coupled with the COVID‐19 pandemic and the Beirut port explosion [8]. In this challenging context and a deteriorated healthcare system, patients might not have the financial means to seek medical advice/counseling from a physician. Amid all this turmoil and the direst of situations, pharmacists continued to serve their people: community pharmacies never closed their doors despite the debilitating financial circumstances, the steep shortage of medications, the lack of universal coverage, and the absence of a strategic vision [9].

Medication adherence, which refers to how patients follow their prescribed medication regimens, is one of the most critical aspects of patient care [10]. Non‐adherence can lead to suboptimal treatment outcomes, increased healthcare costs, and a higher risk of complications [11]. Pharmacists play a pivotal role in addressing this challenge [10]. Indeed, medication adherence is a critical factor in successfully managing chronic conditions and preventing acute illnesses. However, it remains a complex and multifaceted challenge. Patients may struggle with adherence for various reasons, including forgetfulness, concerns about side effects, misunderstanding instructions, or financial constraints [12]; this is added to lifestyle difficulties and disease progression that make it harder for them to remember changing or increasing the number of medications [13]. Pharmacists, as accessible healthcare providers, are uniquely positioned to address these issues. Through patient counseling and education, they can clarify medication regimens, highlight the importance of adherence, and discuss potential side effects, ultimately empowering patients to make informed decisions about their treatment [14]. Moreover, pharmacists can identify and mitigate barriers to adherence, such as medication costs, by collaborating with other healthcare providers to find suitable alternatives, particularly in the challenging context of Lebanon. By fostering open communication and trust, pharmacists also help patients navigate the complexities of their medication regimens, contributing to improved health outcomes [15, 16].

The pharmacist‐patient relationship is a crucial facet of the holistic healthcare experience. When patients feel respected, heard, and well‐informed by their pharmacists, they are more likely to develop a profound trust and confidence in their healthcare providers [17]. This sense of trust, in turn, correlates with heightened adherence to medication regimens, a greater propensity to seek advice and clarifications, and, ultimately, superior health outcomes [18]. Patients who derive efficient relations from their pharmacy experiences also tend to engage in constructive dialogs with their pharmacists, facilitating the early identification and resolution of potential medication‐related problems and concerns [19]. The patient experience with pharmacy visits transcends the effectiveness of medications, encompassing the quality of care and support received throughout the healthcare journey [20]. Pharmacists wield substantial influence in shaping the patient‐pharmacist experience due to their accessibility, proficiency, and the interpersonal relationships they cultivate with their patients. A satisfied patient is more inclined to engage in open and transparent communication, adhere rigorously to treatment recommendations, and consequently reap the benefits of improved health outcomes [21, 22].

While pharmacists undoubtedly exert considerable influence over medication adherence and the patient experience with healthcare, a complex interplay of factors should also be considered. These factors encompass the sociodemographic characteristics of patients, the pharmacist factors, and the nature of the pharmacist‐patient relationship [23, 24]. Patients' age, educational background, socioeconomic status, and health literacy can significantly impact the patient's willingness to adhere to prescribed medications [23]. On the other hand, pharmacist characteristics, including communication skills, empathy, and cultural competence, profoundly affect patient trust and experience [25]. Previous work based on the same database found that patient characteristics, such as age, education, health coverage, and expectations, in addition to services offered by community pharmacists, significantly affected patient satisfaction in times of crisis [26], although no mediation role for pharmacists was found between patients' health status and lack of medication adherence [27]. In light of these contradictory results, and since satisfaction is expected to be linked to medication adherence [21, 22], the authors took a closer look to further depict the detailed factors associated with adherence, including overall satisfaction and other patient‐ and pharmacy‐related items. This study would serve to assess the association of patient characteristics and detailed aspects of community pharmacy experience with patient medication adherence in the Lebanese context.

## 2 Methods

### 2.1 Study Design

This investigation was embedded within the broader IMPHACT‐LB Project, which examines the Impact of the Modern Pharmacy Concept on patient therapy in Lebanon [26, 27]. A total of 865 Lebanese adults completed an online survey between April 11 and April 27, 2023. The web‐based, cross‐sectional design was chosen to efficiently capture responses from a wide and geographically varied population within a short period. Such a design was well‐suited to the study's purpose of exploring associations among patient characteristics, pharmacy encounters, and medication adherence at a single time point, rather than following longitudinal changes.

The questionnaire was created using Google Forms (https://forms.gle/c6gdZf76MSTFzcDN7) and disseminated via multiple social media platforms (WhatsApp, Facebook, and Instagram), enabling participation from individuals across Lebanon, including Beirut, Mount Lebanon, the North, the South, and the Beqaa. Eligibility was restricted to Lebanese residents aged 18 years and above.

At the start of the survey, participants were provided with information on the study objectives and estimated completion time. They were clearly advised that participation was voluntary and that withdrawal was possible at any time. Electronic informed consent was required before proceeding, and no incentives were offered. Participants were not involved in the development, implementation, or dissemination of the study. The study was designed and reported in accordance with the Strengthening the Reporting of Observational Studies in Epidemiology (STROBE) guidelines.

### 2.2 Ethical Aspects

The study protocol was reviewed and approved by the Ethics and Research Committee of the School of Pharmacy at the Lebanese International University (Approval ID: 2023RC‐013‐LIUSOP). All procedures were carried out in line with the ethical standards of the Helsinki Declaration. To safeguard confidentiality, the survey was designed to collect no identifying information, and responses were submitted anonymously. Informed consent was obtained electronically prior to participation. Engagement in the study was entirely voluntary, and no compensation was provided.

### 2.3 Survey Instrument

The questionnaire was administered in Arabic, the official language of Lebanon, and organized into three main parts. The first part gathered sociodemographic details, such as age, educational attainment, employment status, and household income. It also addressed health‐related factors, including existing medical conditions, health insurance coverage, accessibility of healthcare services, and common reasons for visiting a pharmacy.

The second part focused on patterns of community pharmacy use and patients' experiences. Items covered the range of services offered by pharmacists, patients' perceptions and satisfaction with these services, frequency of pharmacy visits, and whether participants regularly attended a particular pharmacy [28, 29].

The third section included the following validated tools:

#### 2.3.1 The Lebanese Medication Adherence Scale (LMAS‐14)

The Lebanese Medication Adherence Scale (LMAS‐14) was used to assess medication adherence. This generic scale was validated in the Lebanese population among patients with cardiovascular [30], cerebrovascular [31], diabetes [32], and thyroid [33] problems. It includes 14 items categorized into three domains linked to psychological, forgetfulness, and economic factors. Each item is graded on a 4‐point scale ranging from “always” to “never.” All items are summed to calculate the overall score, with higher values denoting better medication adherence. As for psychometric properties, sampling adequacy based on factor analysis structure and reliability measures in this study were excellent: Kaiser–Meyer–Olkin (KMO) = 0.932; Barlett's test of sphericity *p* < 0.001; Cronbach's alpha = 0.927.

#### 2.3.2 The InCharge Financial Distress/Financial Well‐Being Scale (IFDFW)

The InCharge Financial Distress/Financial Well‐Being Scale (IFDFW), already validated in the Lebanese population, is an 8‐item subjective assessment of financial distress and well‐being. It was used to evaluate financial distress and the impact of economic hardship [34]. Responses are graded from 1 to 10, with higher scores indicating better financial well‐being [35]. As for psychometric properties, sample adequacy based on factor analysis structure and reliability measures in this study were excellent: KMO = 0.940; Bartlett's test of sphericity *p* < 0.001; Cronbach's alpha = 0.944.

#### 2.3.3 The Adapted Version of the Patient Satisfaction Questionnaire Short Form (Ph‐PSQ‐18)

The Patient Satisfaction Questionnaire Short Form (PSQ‐18) is a concise, validated tool designed to evaluate patient satisfaction with healthcare providers. This 18‐item scale encompasses seven key dimensions of patient satisfaction: General Satisfaction (items 3 and 17), Technical Quality (items 2, 4, 6, and 14), Interpersonal Manner (items 10 and 11), Communication (items 1 and 13), Financial Aspects (items 5 and 7), Time Spent with Doctor (items 12 and 15), and Accessibility and Convenience (items 8, 9, 16, and 18) [36]. Subscale scores were calculated by averaging the responses to items within each dimension, with higher scores indicating greater satisfaction with medical care. For this particular study, the PSQ‐18 was adapted to assess patient satisfaction with pharmacists and community pharmacy services. The adapted version, called Ph‐PSQ‐18, was translated into Arabic through a rigorous process: Initial translation by one of the authors, followed by a review by a second author to identify potential discrepancies or nuances, and the final version agreed upon by all authors, ensuring accuracy and consistency in capturing intended concepts across linguistic and cultural contexts. The Ph‐PSQ‐18 demonstrated excellent psychometric properties: sampling adequacy based on the factor analysis structure (Kaiser–Meyer–Olkin value of 0.966 and Bartlett's test of Sphericity *p*‐value < 0.001), and reliability (Cronbach's alpha of 0.945). These measures indicate that the Ph‐PSQ‐18 is a robust and reliable tool for assessing patient satisfaction with pharmacist services in Arabic‐speaking populations. Further details are presented in a previous publication [26].

### 2.4 Sample Size Calculation

Sample size estimation was first performed using G*Power software (version 3.0.10). With an assumed squared multiple correlation of 0.05 (R² deviation from 0) and an effect size of 0.0526, the calculation for an Omnibus multiple regression test with 25 predictors, an alpha error of 5%, and a power of 80% indicated a minimum requirement of 454 participants.

A second calculation was performed based on adherence prevalence reported in the most recent Lebanese study [37], which represented the main outcome variable. Using Epi Info software (CDC, Atlanta) and setting *α* = 0.05 and *β* = 0.20, the estimated minimum sample size was 384 participants, sufficient to detect a 50% prevalence of adherence with a 5% margin of error.

To satisfy both estimations and account for potential missing data, the study aimed to recruit at least 600 participants.

### 2.5 Statistical Analysis

Data analysis was conducted using IBM SPSS Statistics software, version 28.0. Descriptive statistics included frequencies and percentages for categorical variables, while continuous variables were summarized using means and standard deviations. Normality of distribution was assessed through histogram inspection and confirmed by skewness and kurtosis values below 1. Given the large sample size ( > 300), these criteria were considered consistent with normality assumptions.

To evaluate the psychometric performance of the applied scales, factor analysis was carried out. Sampling adequacy was assessed using the Kaiser–Meyer–Olkin (KMO) index, and Bartlett's test of sphericity was examined. Internal consistency was measured using Cronbach's alpha coefficients.

For bivariate analyses, independent sample *t*‐tests were used to compare means between two groups, while one‐way ANOVA was applied for comparisons across three or more groups. Levene's test assessed homogeneity of variances; when this assumption was not met, corrected *t*‐tests or the Kruskal–Wallis test were applied. Associations between continuous variables were examined using Pearson's correlation coefficient (*r*). A *p*‐value < 0.05 was considered statistically significant.

To identify predictors of medication adherence (measured using the LMAS‐14 score), three linear regression models were constructed using a backward stepwise method. The first model included sociodemographic factors with a *p*‐value < 0.20 in the bivariate analysis. The second model added overall satisfaction, and the third incorporated detailed pharmacist‐related variables alongside the sociodemographic predictors. Regression outputs were presented as unadjusted beta coefficients with 95% confidence intervals (CI). Statistical significance was set at *p* < 0.05 with a 5% margin of error.

## 3 Results

### 3.1 Sample Description

A total of 865 individuals participated in this study. More than half of the participants were females (68.8%), unmarried (60.1%), unemployed (51.1%), with a monthly income of more than 150 US$ (52.5%). Most participants (78.3%) had a university education level, while 45.9% lived in Beirut and Mount Lebanon. The mean age of the participants was 32.52 ± 14.56 years. Table 1 presents the sociodemographic characteristics of the participants.

**TABLE 1:** The sociodemographic and medical characteristics of participants (*n* = 865).

Variable	*N* (%)
Gender	
Male	270 (31.2)
Female	595 (68.8)
Area of residence	
Beirut	215 (24.9)
Mount Lebanon	182 (21.0)
North	151 (17.5)
Beqaa	17 (2.0)
South	300 (34.7)
Marital status	
Unmarried (single/widowed/divorced)	520 (60.1)
Married	345 (39.9)
Monthly income	
Less than 4,000,000 LBP*	44 (5.1)
Between 4,000,000 and 8,000,000 LBP*	176 (20.3)
Between 8,000,000 and 12,000,000 LBP*	191 (22.1)
More than 12,000,000 LBP*	454 (52.5)
Education level	
Illiterate	16 (1.8)
School level	172 (19.9)
University level	677 (78.3)
Employment status	
Unemployed	442 (51.1)
Employed	407 (47.1)
Retired	16 (1.8)
Declared health status	
No sickness	686 (79.3)
Chronic medical conditions	169 (19.5)
Severe sickness	10 (1.2)
Presence of a medical illness**	
Hypertension	97 (11.2)
Diabetes mellitus	58 (6.7)
Dyslipidemia	85 (9.8)
Cardiovascular diseases	51 (5.9)
Chronic kidney disease	21 (2.4)
Asthma	51 (5.9)
COPD*	10 (1.2)
Other chronic pulmonary diseases	13 (1.5)
Allergic conditions	217 (25.1)
History of stroke or cerebrovascular diseases	19 (2.2)
Seizures or other neurological conditions	37 (4.3)
Depression, anxiety, or other psychiatric conditions	122 (14.1)
Chronic gastrointestinal conditions	122 (14.1)
Current or previous cancer	17 (2.0)
Rheumatic disease	43 (5.0)
Other chronic diseases	51 (5.9)
Easy access to healthcare	
Yes	671 (77.6)
No	194 (22.4)
Health coverage**	
Private insurance	253 (29.2)
National Social Security Fund	303 (35.0)
Ministry of Public Health	52 (6.0)
Self‐payer	175 (20.2)
Public insurance (Army, COOP, Internal Security Forces)	129 (14.9)
Irregular coverage/NGOs and PHC	88 (10.2)
	Mean ± SD
Age (years)	32.52 ± 14.56 [Min 18; Max 88]
IFDFW score*	39.88 ± 18.11 [Min 8; Max 80]
Ph‐PSQ18 score	53.36 ± 2.58 [Min 41; Max 65]
LMAS‐14 score	34.15 ± 11.23 [Min 14; Max 56]
Current family size	4.78 ± 2.40 [Min 0; Max 43]
Number of chronic diseases	1.42 ± 2.16 [Min 0; Max 16]
Number of medications taken routinely per day	0.87 ± 1.78 [Min 0; Max 17]

* COPD, Chronic Obstructive Pulmonary Disease; LBP, Lebanese Pounds; 90,000LBP are equivalent to 1 US $; IFDFW, The InCharge Financial Distress/Financial Well‐Being Scale; LMAS, Lebanese Medication Adherence Scale; Ph‐PSQ18, Pharmacy Related Patient Satisfaction Questionnaire.

** Each participant might have multiple answers.

### 3.2 Description of the Health Variables and LAMS‐14 Score Distribution

Furthermore, in Table 1, 19.5% had chronic medical diseases, 25.1% had allergic problems, 14.1% had depression, anxiety, or other psychiatric disorders, and 14.1% had chronic gastrointestinal conditions. The majority (77.6%) declared having easy access to healthcare, and only 29.2% had private insurance. The mean number of chronic diseases was 1.42 ± 2.16 (15% declared having at least one chronic disease), and the mean number of daily medications was 0.87 ± 1.78 (33% were taking at least one medication).

The Ph‐PSQ18 averaged 53.36 ± 2.58. As for the LMAS‐14, the reported average was 34.15 ± 11.23, divided into 4 quartiles: 25.5% below 17, 24.5% between 27 and 33, 27.6% between 34 and 42, and 22.3% above 42.

### 3.3 Description of Community Pharmacy Visits and Related Experience

Of all participants, 38.5% were regular visitors to the community pharmacy, 57.7% visited the community pharmacy to obtain nonprescription medications, 10.6% visited the community pharmacy weekly for medical care or counseling, and 50.9% visited the same pharmacy each time. Among those who stated always visiting the community pharmacy, 8.2% visited the community pharmacy to obtain nonprescription medications, 3.9% sought initial medical assessment and/or care, 4.9% visited the community pharmacy for general or specific medical advice, and 9.9% preferred discussing medical conditions with the pharmacist. More than half of the participants came to a community pharmacy to ask about medications (68.2%), while 36.0% came to ask about diseases, and 45.8% asked for treatment from the pharmacist. Almost half of the participants reported that the counseling time by the pharmacist was 5–10 min (47.6%), 50.8% sought counseling by the pharmacist from time to time, and 33.2% stated that the pharmacy has a counseling area. The majority of the participants perceived the pharmacist as a medication expert (94.2%), a health counselor (81.0%), a health promoter (66.2%), and a patient‐centered practitioner (73.3%). Table 2 provides an overview of community pharmacy visits and the associated experiences.

**TABLE 2:** Description of community pharmacy visits and related experience.

Variable	*N* (%)
Familiar with the current community pharmacy	
Recent visitor (< 1 year)	192 (22.2)
Regular visitor (1–5 years)	333 (38.5)
Chronic visitor (> 5 years)	340 (39.3)
A major reason for visiting a community pharmacy	
Obtain chronic medications (hypertension, diabetes mellitus, cardiovascular, etc.)	81 (9.4)
Obtain nonprescription medications (analgesics, anti‐inflammatory, supplements, etc.)	499 (57.7)
Obtain both chronic and nonprescription medications	152 (17.6)
Other reason	133 (15.4)
Visit the same pharmacy each time.	
Yes, the same pharmacy each time	440 (50.9)
No, different pharmacies depending on the situation/circumstances	425 (49.1)
Visiting a community pharmacy for medical care or counseling	
Daily	16 (1.8)
Weekly	92 (10.6)
Monthly	349 (40.3)
Rarely	408 (47.2)
Visiting your community pharmacy to obtain nonprescription medications	
Always	71 (8.2)
Most of the time	283 (32.7)
Sometimes	169 (19.5)
Rarely	302 (34.9)
Never	40 (4.6)
Visiting your community pharmacy to seek initial medical assessment and/or care	
Always	34 (3.9)
Most of the time	168 (19.4)
Sometimes	259 (29.9)
Rarely	284 (32.8)
Never	120 (13.9)
Visiting your community pharmacy for general or specific medical advice	
Always	42 (4.9)
Most of the time	173 (20.0)
Sometimes	258 (29.8)
Rarely	279 (32.3)
Never	113 (13.1)
Do you prefer discussing your medical conditions with your pharmacist before referring to your primary care provider or doctor?	
Always	86 (9.9)
Most of the time	229 (26.5)
Sometimes	204 (23.6)
Rarely	241 (27.9)
Never	105 (12.1)
The pharmacy that you visit includes a counseling area.	
I do not know	269 (31.1)
No	309 (35.7)
Yes	287 (33.2)
Mainly, I come to a community pharmacy to ask about medications.	
No	275 (31.8)
Yes	590 (68.2)
Mainly, I come to a community pharmacy to ask about a disease.	
No	554 (64.0)
Yes	311 (36.0)
Mainly, they come to a community pharmacy to receive treatment from the pharmacist	
No	469 (54.2)
Yes	396 (45.8)
Receiving regular counseling from your pharmacist	
No, not at all	252 (29.1)
Yes, regularly	174 (20.1)
Yes, from time to time	439 (50.8)
How long does the pharmacist spend counseling you about a medication and/or medical condition?	
Less than 5 min	372 (45.8)
5–10 min	387 (47.6)
More than 10 min	54 (6.6)
Perceive your pharmacist as a medication expert.	
No	50 (5.8)
Yes	815 (94.2)
Perceive your pharmacist as a health counselor	
No	164 (19.0)
Yes	701 (81.0)
Perceive your pharmacist as a health promoter	
No	292 (33.8)
Yes	573 (66.2)
Perceive your pharmacist as a patient‐centered practitioner	
No	231 (26.7)
Yes	634 (73.3)
Your pharmacist advises you about healthy lifestyle modifications, including smoking cessation, weight loss, or physical activity.	
No	244 (28.2)
Yes	621 (71.8)
Pharmacists provide additional healthcare services like measuring blood pressure and/or glucose.	
No	175 (20.2)
Yes	690 (79.8)

### 3.4 Bivariate Analysis

Table 3 presents the bivariate analysis with the LMAS‐14 score as the dependent variable. The results showed that the mean LMAS‐14 score was significantly higher among participants with public insurance than among those in all other categories (36.12 vs. 33.80, *p* = 0.030), while those with irregular coverage had a significantly lower mean of the LMAS‐14 score (31.26 vs. 34.47, *p* = 0.003). Older age was significantly associated with a higher LMAS‐14 score (*r* = 0.097, *p* = 0.004). All other variables had no significant association with LMAS‐14 (*p* > 0.05), including patient overall satisfaction with pharmacy experience.

**TABLE 3:** Bivariate analysis taking the LMAS‐14 score as the dependent variable.

Variable	LMAS‐14 score		*p* value
Gender			
Male	35.12 ± 11.70		0.087
Female	33.70 ± 10.99		
Marital status			
Unmarried (single/widowed/divorced)	33.56 ± 10.87		0.062
Married	35.02 ± 11.71		
Monthly income			
Less than 4,000,000 LBP*	34.22 ± 11.09		0.984
Between 4,000,000 and 8,000,000 LBP*	34.36 ± 10.43		
Between 8,000,000 and 12,000,000 LBP*	33.90 ± 10.29		
More than 12,000,000 LBP*	34.16 ± 11.93		
Education level			
School level	34.54 ± 11.39		0.589
University level	34.04 ± 11.19		
Work status			
Unemployed	33.87 ± 11.23		0.439
Employed	34.46 ± 11.23		
Health status			
No sickness	33.87 ± 11.17		0.162
Medical conditions	35.19 ± 11.41		
Easy access to healthcare			
Yes	33.78 ± 11.41		0.072
No	35.42 ± 10.50		
Health coverage			
Private insurance	Yes	34.24 ± 11.62	0.868
	No	34.10 ± 11.07	
National Social Security Fund	Yes	33.26 ± 11.30	0.090
	No	34.62 ± 11.17	
Ministry of Public Health	Yes	35.78 ± 11.05	0.278
	No	34.04 ± 11.24	
Self‐payer	Yes	34.62 ± 10.22	0.500
	No	34.02 ± 11.47	
Public insurance (Army, COOP, Internal Security Forces)	Yes	36.12 ± 11.31	**0.030**
	No	33.80 ± 11.19	
Irregular coverage, such as NGOs and PHC	Yes	31.26 ± 9.10	**0.003**
	No	34.47 ± 11.40	
The main reason for visiting a community pharmacy			
Obtain chronic medications (hypertension, diabetes mellitus, cardiovascular, etc.)	35.35 ± 12.35		0.072
Obtain nonprescription medications (analgesics, anti‐inflammatory, supplements, etc.)	34.29 ± 11.17		
Obtain both chronic and nonprescription medications	32.13 ± 11.21		
Other reason	35.16 ± 10.56		
The frequence of visits to your community pharmacy to obtain nonprescription medications			
Always	37.38 ± 11.82		0.095
Most of the time	33.99 ± 10.61		
Sometimes	33.79 ± 11.72		
Rarely	34.04 ± 11.22		
Never	31.77 ± 11.79		
The amount of time the pharmacist spends counseling you about a medication and/or a medical condition:			
Less than 5 min	33.92 ± 11.06		0.094
5–10 min	33.96 ± 11.37		
More than 10 min	37.38 ± 11.19		
	Pearson correlation coefficient		
Age	0.097		**0.004**
IFDFW* score	0.023		0.502
Overall satisfaction score (Ph‐PSQ18)*	−0.036		0.293
Number of chronic diseases	0.016		0.629
Number of medications taken routinely per day	0.009		0.781

*Note:* Bold values are statistically significant at *p* < 0.05.

**Each participant might have multiple answers.

* LBP, Lebanese Pounds; IFDFW, The InCharge Financial Distress/Financial Well‐Being Scale; Ph‐PSQ18, Pharmacist‐Patient Satisfaction Questionnaire 18.

### 3.5 Multivariable Analysis

A first linear regression model taking the LMAS‐14 score as the dependent variable and the sociodemographic variables as independent variables showed that older age (Beta = 0.06) and having public insurance (Beta = 2.11) were significantly associated with a higher LMAS‐14 score. However, having irregular health coverage (Beta = −2.92) was significantly associated with a lower LMAS‐14 score (Table 4, Model 1).

**TABLE 4:** Multivariable linear regression analysis taking the LMAS‐14 score as the dependent variable.

	Unstandardized Beta	Standardized Beta	*p* value	95% Confidence Interval	
				Lower bound	Upper bound
Model 1: Linear regression analysis taking the LMAS‐14 score as the dependent variable and the sociodemographic characteristics of participants as independent variables**					
Age	0.067	0.087	0.010	0.016	0.119
Health coverage irregular coverage (yes vs. no)*	−2.929	−0.079	0.020	−5.395	−0.464
Health coverage public insurance (yes vs. no)*	2.112	0.067	0.048	0.021	4.202
Model 2: Linear regression analysis taking the LMAS‐14 score as the dependent variable and the sociodemographic characteristics and overall satisfaction with community pharmacy experience of participants as independent variables**					
Lower bound	Upper bound
Total satisfaction from community pharmacy experience	−0.135	−0.031	0.361	−0.423	0.154
Health coverage public insurance (yes vs. no)*	2.093	0.066	0.050	0.003	4.184
Health coverage irregular coverage (yes vs. no)*	−2.908	−0.078	0.021	−5.374	−0.442
Age	0.067	0.087	0.011	0.016	0.118
Model 3: Linear regression analysis taking the LMAS‐14 score as the dependent variable and the sociodemographic characteristics and community pharmacy experience variables as independent variables***					
Age	0.081	0.106	0.003	0.028	0.134
Reason for visiting the pharmacy obtain both chronic and nonprescription medications	−2.981	−0.102	0.004	−5.003	−0.958
Visit your community pharmacy to obtain nonprescription medications (always vs. never)*	3.630	0.090	0.009	0.902	6.359
Health coverage public insurance (yes vs. no)*	2.374	0.075	0.030	0.236	4.512
Health coverage irregular coverage (yes vs. no)*	−2.604	−0.070	0.043	−5.120	−0.088
Pharmacists spend more on counseling you about a medication and/or medical condition (more than 10 min vs. less than 5 min)*	3.105	0.069	0.046	0.051	6.160

* Reference category.

** Variables initially entered in the model: gender, marital status, health status, easy access to healthcare, health coverage, and age.

*** Variables entered in the model: gender, marital status, health status, health coverage, age, reason for visiting community pharmacy, visiting community pharmacy to obtain nonprescription medications, and the amount of time the pharmacist spends on counseling you about a medication and/or medical condition.

A second linear regression was performed, adding overall patient satisfaction from the community pharmacy experience to the model as an independent variable; patient satisfaction was not significantly associated with medication adherence (*p* > 0.05) (Table 4, Model 2).

A third linear regression model was performed by adding the pharmacist‐related variables to the first model as independent variables. Results showed that older age (Beta = 0.08), always visiting the community pharmacy to obtain nonprescription medications (Beta = 3.63), having public health coverage (Beta = 2.37), and receiving more than 10 min of counseling about a medication and/or medical condition (Beta = 3.10) were significantly associated with a higher LMAS‐14 score. Visiting a pharmacy to obtain both chronic and nonprescription medications (Beta = −2.98) and having irregular health coverage (Beta = −2.60) were significantly associated with a lower LMAS‐14 score (Table 4, Model 3).

## 4 Discussion

The present study assessed patients' characteristics, their community pharmacy experience, and the association of these factors with adherence to prescribed medications. Among the key findings, it was observed that fewer than half of the participants were regular visitors to community pharmacies, with approximately half of them consistently visiting the same pharmacy. Primary reasons for visiting pharmacies were obtaining nonprescription medications, seeking information about medications, inquiring about specific diseases, and receiving treatment from pharmacists. Moreover, findings indicated that most of the population holds pharmacists in high regard, perceiving them as experts in medications, health counselors, health promoters, and patient‐centered healthcare practitioners.

The current findings also revealed a positive association between patient medication adherence and factors, including older age and public health insurance; no association was found with pharmacy‐related overall satisfaction. However, regular pharmacy visits for nonprescription medications, and receiving counseling from the pharmacist for more than 10 min showed a positive association with medication adherence. Conversely, lower medication adherence was associated with visiting pharmacies for both chronic and nonprescription medications and having irregular health insurance coverage.

This study highlights the diverse reasons for patient visits to community pharmacies, including medical care, advice, and discussing medical conditions with pharmacists. These results are consistent with the evolving role of pharmacists as healthcare providers, emphasizing patient‐centered care and expanded clinical services [38, 39, 40]. However, the relatively short counseling time reported by some participants suggests potential areas for improvement in the community pharmacy experience. It is worth mentioning that the relatively short counseling duration may be attributed to factors such as pharmacist fatigue and reduced productivity resulting from sickness and depression. Earlier research conducted in Lebanon has indicated that a significant portion of community pharmacists experience emotional, mental, and physical fatigue at work, along with work‐related productivity challenges due to illness and depression [41, 42]. This study also found that a considerable proportion of participants visit community pharmacies for nonprescription medications, which lines up with the role of these pharmacies as accessible sources for over‐the‐counter medications [43]. This result aligns with previous findings indicating that community pharmacies are crucial in managing minor ailments and providing convenient access to healthcare products [44, 45].

Moreover, the prevailing perception of pharmacists as medication experts, health counselors, health promoters, and patient‐centered practitioners, as revealed in this study, aligns with a growing body of research in pharmacy practice. It is essential to highlight that these positive perceptions play a pivotal role in shaping the community pharmacy experience, with ultimately profound implications for patient care. According to the International Pharmaceutical Federation (FIP), community pharmacists are recognized as medication specialists with expertise in several areas, such as medication assessment, compounding, dispensing, providing information and advice, and monitoring, among various other competencies [46]. Numerous studies support the current findings, demonstrating that pharmacists are increasingly considered essential healthcare providers. For instance, a recent study revealed that the general population perceives pharmacists as trusted and valuable healthcare providers and medication experts [17]. Similarly, another study found that patients perceive pharmacists as knowledgeable healthcare professionals who can provide beneficial guidance on health‐ and medication‐related issues [47]. Notably, a survey conducted in Kuwait revealed that 96% of physicians perceive pharmacists as integral members of the healthcare team, and 92% believe pharmacists possess additive value to the patient care process [48].

Nonetheless, the current study found that less than half of the participants were regular visitors to community pharmacies. Although the reason for this finding is not fully understood, it is hypothesized that the ongoing socioeconomic crisis in Lebanon may have reduced the public's ability to acquire nonprescription medications for minor ailments and other products considered less essential [8]. Furthermore, the participants in the current study were relatively young, with an average age of 32.52 years. This demographic skew toward youth may be linked to lower illness and reduced healthcare and medication requirements. Additional research is suggested to delve deeper into this observation and its implications.

Medication adherence is critical to achieving optimal healthcare outcomes [49]. In line with the findings of previous research examining medication adherence among Lebanese patients with diverse medical conditions [30, 31, 32], the results of the current study demonstrated a satisfactory level of adherence, with several sociodemographic and pharmacist‐related variables significantly predicting this adherence. Older age was positively associated with better medication adherence. Although the sample in this study was relatively young, older individuals often have more experience managing chronic conditions and a greater understanding of the importance of medication adherence. This finding is consistent with several published studies that observed a positive relationship between age and adherence to prescribed medications [50, 51].

Having public insurance was also significantly associated with better medication adherence. Conversely, having irregular health coverage was associated with lower adherence, highlighting the role of health coverage in medication adherence. Public insurance programs often provide better access to healthcare services, covering medications and reducing financial barriers that hinder adherence [52]. On the other hand, irregular health coverage options, such as non‐governmental organizations or primary healthcare centers, are generally linked to a lower socio‐economic status, found in vulnerable populations who have fewer resources and access to medication, thus disrupting medication adherence [53].

The results from this study highlight the significance of pharmacist‐related variables as predictors of patient medication adherence, showing a discrepancy between overall patient satisfaction and detailed pharmacy‐related experience aspects. Indeed, overall pharmacy‐related patient satisfaction was not associated with medication adherence: the satisfaction concept included diverse aspects, such as General Satisfaction, Technical Quality, Interpersonal Manner, Communication, Financial Aspects, Time Spent with the Pharmacist, Accessibility, and Convenience [36]. None of these aspects was associated with medication adherence (results not shown). Nevertheless, visiting the community pharmacy regularly for nonprescription medications and receiving more than 10 min of counseling from the pharmacist were significantly associated with better medication adherence. Conversely, visiting the pharmacy for chronic and nonprescription medications was associated with lower adherence. These results show that patients have different adherence behaviors according to the condition, and pharmacists in Lebanon might have differential behaviors when dispensing chronic or acute medications, taking maybe more time to explain new medications to the patients (11% vs. 5.6% of patients declaring more than 10‐min counseling time; *p* = 0.06), thus improving their adherence. This particular aspect of the patient‐pharmacist relationship deserves further longitudinal studies, although it has been inferred based on some publications [54, 55].

Thus, the positive impact of pharmacist‐related variables still aligns with the role of pharmacists in patient care, emphasizing the importance of patient education and counseling [56]. Spending more time with patients, especially when discussing medications and medical conditions, can enhance their understanding and adherence [57]. In contrast, the negative association with visiting the pharmacy for chronic and nonprescription medications may suggest potential distractions or time constraints affecting counseling opportunities [58], which may necessitate increased efforts by pharmacists to offer extensive counseling to these patients and pinpoint additional factors linked to their lower adherence, especially considering that this patient subgroup needs more in‐depth counseling and may be at a higher risk of developing subsequent health complications.

### 4.1 Implications for Practice

The results of the current study carry several practical implications for pharmacy practice. First, the findings emphasize the importance of pharmacists paying extra attention to medication adherence among younger individuals, as this group appears more prone to lower adherence.

Second, pharmacists may play a vital role in identifying affordable alternatives and interventions to enhance medication adherence for patients with certain types of insurance that do not cover medications.

Third, the need for pharmacists to provide more extensive counseling, especially for those with chronic conditions, is underscored, as spending more time on counseling about medications and medical conditions was associated with improved adherence, suggesting that increased counseling efforts could help reduce counseling‐related non‐adherence, particularly for chronic diseases.

These insights can guide pharmacists in tailoring their services to better support patients in various age groups, insurance categories, and counseling needs, ultimately improving medication adherence and health outcomes.

Lastly, the findings from this study reveal potential areas for collaboration between pharmacists and other healthcare providers to improve patient medication adherence. These areas include finding more affordable alternative medications when necessary and expanding patient education and health promotion efforts. In summary, as recommended by the World Health Organization [49], it is necessary to improve patient education and involvement in treatment decisions, simplify medication regimens, enhance healthcare provider communication and follow‐up, address socioeconomic barriers to medication access, and provide support for managing side effects.

### 4.2 Limitations and Strengths

This study has several limitations. First, the cross‐sectional design employed in the study cannot establish temporal relationships, preventing confirmation of causality. Second, the sampling method may have skewed the sample toward a higher proportion of younger individuals with higher education levels and fewer chronic health conditions. Moreover, because recruitment relied on digital platforms, older adults and individuals with limited internet access or digital literacy may have been underrepresented, further constraining the representativeness of the sample. Additionally, the sample displayed an overrepresentation of females compared to males, potentially influencing the community pharmacy experience and medication adherence patterns.

Furthermore, the reliance on self‐reported data introduces a possible risk of information bias, although this risk is deemed minimal and unlikely to exhibit differential associations. Lastly, despite conducting multivariable analyses on medication adherence, residual confounding related to specific health literacy may persist.

Nevertheless, the study carries many strengths. It included a diverse sample drawn from various districts across Lebanon, encompassing individuals from varying financial backgrounds, potentially enhancing the generalizability of the findings. Moreover, the sample size was sufficient for conducting all statistical analyses with adequate power, precluding potential confounding factors.

Further research endeavors are warranted to address and mitigate the acknowledged limitations and further corroborate the results. According to the World Health Organization [49], the following factors are essential and should be considered for medication adherence: socioeconomic factors, healthcare team and system‐related factors, condition‐related factors, therapy‐related factors, and patient‐related factors. Thus, further longitudinal studies that detail the suggested factors are recommended.

## 5 Conclusion

This study highlighted the sociodemographic attributes and aspects of the community pharmacy experience that influence patient medication adherence. Pharmacists are widely perceived as medication experts, health counselors, health promoters, and patient‐centered healthcare practitioners. Recognizing the impact of age and insurance coverage on adherence can inform targeted interventions to support individuals at higher risk of non‐adherence. Furthermore, emphasizing the role of pharmacists in patient care, mainly through extended counseling sessions, can contribute to improved medication adherence and, consequently, better health outcomes.

## Author Contributions

All authors contributed to the study's conception and design. Fouad Sakr, Hala Sacre, Marwan Akel, Aline Hajj, Rony Zeenny, Pascale Salameh, and Jihan Safwan prepared the material and collected the data. Chadia Haddad analyzed the data under the supervision of Pascale Salameh. Fouad Sakr drafted the first version of the manuscript. All authors have read and approved the final version of the manuscript. Fouad Sakr and Pascale Salameh had full access to all of the data in this study and take complete responsibility for the integrity of the data and the accuracy of the data analysis.

## Funding

The authors received no specific funding for this work.

## Disclosure

The lead author Jihan Safwan affirms that this manuscript is an honest, accurate, and transparent account of the study being reported; that no important aspects of the study have been omitted; and that any discrepancies from the study as planned (and, if relevant, registered) have been explained.

## Ethics Statement

The Ethics and Research Committee of the School of Pharmacy at the Lebanese International University approved the project (Approval number: 2023RC‐013‐LIUSOP). The study adhered to the ethical principles and guidelines outlined in the Helsinki Declaration throughout its execution.

## Consent

Before enrolling in the survey, informed consent was obtained from all participants. Participation was voluntary, and respondents received no incentive in return.

## Conflicts of Interest

The authors declare no conflicts of interest.

## Data Availability

The data that support the findings of this study are available from the corresponding author upon reasonable request.
